# Increased Food Intake by Insufficient Sleep in Humans: Are We Jumping the Gun on the Hormonal Explanation?

**DOI:** 10.3389/fendo.2014.00116

**Published:** 2014-07-15

**Authors:** Jean-Philippe Chaput, Marie-Pierre St-Onge

**Affiliations:** ^1^Healthy Active Living and Obesity Research Group, Children’s Hospital of Eastern Ontario Research Institute, Ottawa, ON, Canada; ^2^Department of Medicine, College of Physicians and Surgeons, New York Obesity Nutrition Research Center, Columbia University, New York, NY, USA

**Keywords:** sleep, food intake, energy balance, obesity, hormones

Concurrent with the increase in obesity prevalence, a decrease in sleep duration has been observed over the past decades (1, 2). Scientists have been intrigued by this inverse trend between sleep and body weight, and studies published in the past 10 years have been helpful in determining if the lack of sleep is a possible cause of obesity (3). Epidemiologic evidence has shown that short sleep duration is associated with obesity and weight gain (4–6), while short-term experimental studies have provided important mechanistic explanations (7–9). More recently, intervention studies have also been able to demonstrate that sleep restriction causes weight gain (10, 11). Collectively, the evidence suggests that insufficient sleep plays a role in the risk of gaining weight, and sleep hygiene should be considered in the prevention as well as in the treatment of obesity (12–14).

The main mechanism by which insufficient sleep may predispose to weight gain is through an increase in food intake (15). In fact, sleep restriction appears to increase 24-h energy expenditure by ~5%, mainly due to the energy cost of additional wakefulness (11, 16). Although a decrease in physical activity energy expenditure is possible for some individuals after sleep restriction, large inter-individual variations exist, and we concluded from a recent comprehensive review of the literature that short sleep duration does not substantially affect components of energy expenditure (17). In contrast, there is strong support for the notion that restricting sleep increases food intake (18–20), suggesting that energy consumption is a key mediator of the association between insufficient sleep and weight gain.

It is well demonstrated that insufficient sleep enhances our vulnerability to overeat in the current obesogenic environment (15, 21). Indeed, there is accumulating and consistent evidence showing that short sleep duration, poor sleep quality, and later bedtimes are all associated with increased food intake, poor diet quality, and excess body weight (15). This field of research has been fueled by the seminal study conducted by Spiegel et al. (22) in which they experimentally tested the acute effects of sleep restriction on appetite control. Briefly, they observed that healthy young men undergoing two nights of sleep restriction with controlled energy intake via an intravenous glucose infusion had decreased leptin levels, elevated ghrelin levels, and increased self-reported ratings of hunger and appetite. However, feeding procedures not typical of real-life conditions in this controlled experiment certainly limit the external generalizability of the findings. Interestingly, experimental studies that have relied on free access to food, a scenario more representative of daily living, are generally consistent in showing that sleep restriction is not associated with changes in ghrelin or leptin levels, or even leads to an increase in leptin levels (23–28). Despite no up-regulation of appetite-stimulating hormones, *ad libitum* experiments are generally consistent in showing that sleep restriction increases caloric intake. Thus, excess energy intake associated with not getting enough sleep appears to be preferentially driven by hedonic rather than hormonal factors (15, 29).

The growing body of evidence not being able to replicate Spiegel’s findings in a more natural context with regard to leptin and ghrelin levels certainly suggests that the “hormonal explanation” is perhaps not the most important mechanism to explain the link between lack of sleep and increased food intake. There are at least two important explanations to this observation. First, sleep timing (bedtime and wake up time) certainly has implications on sleep architecture and appetite hormones (30–34). Early wake times in the sleep restriction condition, as in Spiegel’s study, as opposed to anchoring the sleep episode on wake up time, is likely to stimulate hunger by increasing appetite hormones (probably due to the increased fasting period between wake time and the initial blood draw). Second, the nutritional state and energy balance of study participants can also contribute to explain the conflicting results with regard to leptin and ghrelin levels. While conditions of energy restriction certainly help to enhance the neuroendocrine response of appetite, *ad libitum* conditions, mimicking a state of positive energy balance, are sufficient to explain the absence of difference in leptin and ghrelin concentrations with sleep loss (35, 36).

So, if recent investigations suggest that a change in appetite hormones is not the main mechanism by which sleep restriction increases food intake, what are some potential explanations? As shown in Figure 1, many recent sleep restriction studies suggest that the hedonic aspects of food intake override hormonal factors (11, 26, 28, 37). Eating in the absence of hunger is a common phenomenon in today’s environment characterized by easy access to palatable foods and food intake is proportional to the time spent awake (4, 14). Studies have consistently shown that insufficient sleep increases snacking, the number of meals eaten per day, and the preference for energy-dense food items (15). New neuroimaging experiments have also provided evidence that insufficient sleep enhances hedonic stimulus processing in the brain underlying the drive to consume foods (38–40). In particular, the insular cortex as well as areas thought to be involved in hedonic functions (e.g., orbitofrontal cortex and dorsolateral prefrontal cortex) have been found to display the strongest activation in response to unhealthy food compared to healthy food stimuli after a period of restricted sleep (40).

**Figure 1:**
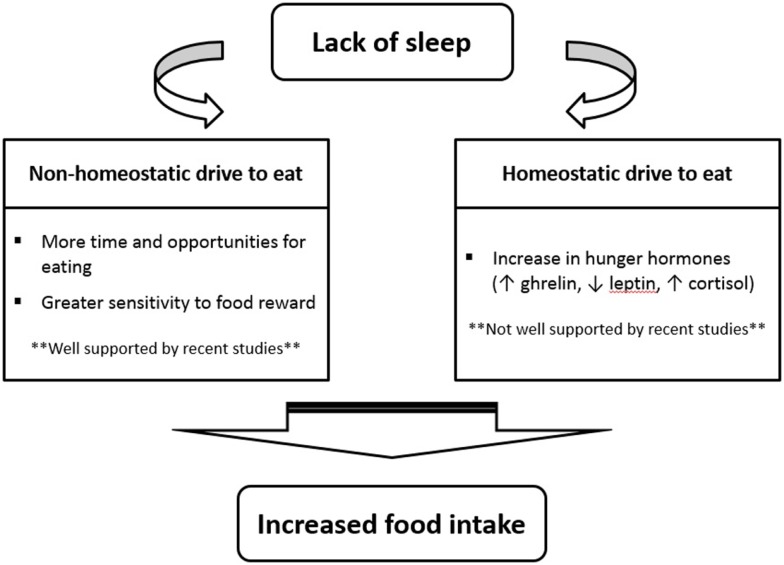
**Proposed pathways by which insufficient sleep increases caloric consumption**.

In conclusion, excess energy intake associated with not getting adequate sleep seems to be preferentially explained by hedonic rather than hormonal factors in the current obesogenic environment. Future studies should focus more on the rewarding aspects of food that accompany sleep loss and scientists should be more cognizant of the factors that affect hormonal measurements in the context of sleep conditions. Those factors include sex, state of energy balance (positive vs. negative), feeding status, timing of sleep and eating episodes, differences in food intake (i.e., diet quality), etc. Understanding the neuronal circuitry and central nervous system regions involved in evoking the hedonic response to food stimuli that accompanies sleep loss will be an active area of investigation in the years to come. The increased ghrelin/decreased leptin hypothesis is much too simplistic when describing the role of sleep duration in the control of food intake. Such an explanation is possibly not the key mediator, and certainly not the sole mediator, of the link between insufficient sleep and obesity.

## Conflict of Interest Statement

The authors declare that the research was conducted in the absence of any commercial or financial relationships that could be construed as a potential conflict of interest.
